# Genome Sequence of Vibrio nigripulchritudo Strain TUMSAT-TG-2018, Isolated from Diseased Pacific White Shrimp, *Litopenaeus vannamei*

**DOI:** 10.1128/MRA.01206-20

**Published:** 2020-12-03

**Authors:** Satoshi Kawato, Jian Lu, Reiko Nozaki, Hidehiro Kondo, Ikuo Hirono

**Affiliations:** a Laboratory of Genome Science, Tokyo University of Marine Science and Technology, Tokyo, Japan; Georgia Institute of Technology

## Abstract

The Gram-negative bacterium Vibrio nigripulchritudo is an important shrimp pathogen. Here, we present the genome sequence of Vibrio nigripulchritudo TUMSAT-TG-2018, which was isolated from a diseased Pacific white shrimp (*Litopenaeus vannamei*). The assembly totaled 6.8 Mbp, consisting of two chromosomes and four plasmids.

## Abstract (precis)

The Gram-negative bacterium Vibrio nigripulchritudo is an important shrimp pathogen. Here, we present the genome sequence of Vibrio nigripulchritudo TUMSAT-TG-2018, which was isolated from a diseased pacific white shrimp (*Litopenaeus vannamei*). The assembly totaled 6.8 Mbp, consisting of two chromosomes and four plasmids.

## ANNOUNCEMENT

The Gram-negative bacterium Vibrio nigripulchritudo is an important shrimp pathogen reported from New Caledonia (1, 2), Madagascar (2), and Japan (3). We observed a mass mortality event involving Pacific white shrimp (*Litopenaeus vannamei*) in a closed seawater aquarium at the Shinagawa Campus, Tokyo University of Marine Science and Technology (Tokyo, Japan), in November 2018. We streaked the muscle of a diseased shrimp onto a heart infusion agar supplemented with 2.5% (wt/vol) NaCl, and *V. nigripulchritudo* TUMSAT-TG-2018 was isolated as a single colony. The outbreak began shortly after cultured kuruma shrimp (*Marsupenaeus japonicus*) purchased from a Japanese farm were introduced into the tank, suggesting that the strain originated from the kuruma shrimp.

Here, we present the genome sequence of *V. nigripulchritudo* TUMSAT-TG-2018. We first sequenced the strain with the Illumina platform to obtain a draft assembly and subsequently performed Nanopore long-read sequencing, using a different DNA preparation, to obtain a chromosome-level assembly. For both preparations, the strain was cultured with heart infusion broth supplemented with 2.5% (wt/vol) NaCl. No shearing or size selection of the extracted DNA was performed before library preparation. Default parameters were used for all software unless otherwise noted.

Genomic DNA for Illumina sequencing was extracted using cetyltrimethylammonium bromide extraction. An Illumina library was prepared with the Nextera XT DNA library preparation kit. A 2 × 150-bp paired-end run with the MiSeq reagent kit v2 yielded 1,568,793 paired-end reads (410.4 Mb). The Illumina reads were quality filtered using Fastp, version 0.20.1 (4), followed by *de novo* assembly using SPAdes, version 3.14.0 (5), with the following settings: careful, only-assembler, k 21,33,55,77,89,101,113,125. The SPAdes draft assembly (6.78 Mbp, with 126 contigs and scaffolds; *N*_50_, 247,739 bp) contained two contigs corresponding to plasmids pVNTG3 and pVNTG4 (Table 1). The two contigs were circularized by manually trimming a 125-bp start-end overlap.

**TABLE 1:** Assembly statistics of the *V. nigripulchritudo* TUMSAT-TG-2018 genome

Contig	GenBank accession no.	Topology	Length (bp)	GC content (%)	No. of^*a*^:				Mean coverage^*b*^ (×) for:	
					CDS	rRNAs	tRNAs	tmRNAs	Illumina reads	Nanopore reads
Chromosome 1	AP024087	Circular	4,072,236	46.06	3,618	31	104	1	56.2	126.4
Chromosome 2	AP024088	Circular	2,160,687	45.54	1,954	0	6	0	49.9	111.2
Plasmid pVNTG1	AP024089	Circular	351,712	44.04	326	0	0	0	52.7	133.0
Plasmid pVNTG2	AP024090	Circular	172,738	41.40	195	0	0	0	76.3	91.7
Plasmid pVNTG3	AP024091	Circular	42,593	42.70	52	0	0	0	418.5	9.8
Plasmid pVNTG4	AP024092	Circular	37,131	41.92	45	0	0	0	515.1	14.3

a CDS, coding sequences; tmRNAs, transfer-messenger RNAs.

b Mean coverage values were calculated using SAMtools coverage, version 1.10 (13), from BAM alignment files generated with minimap2 (8), version 2.17, with the settings ax sr for Illumina and ax map-ont for Nanopore reads.

For Nanopore sequencing, genomic DNA was extracted using a NucleoBond AXG 100 column and the NucleoBond buffer set III (Macherey-Nagel). A long-read library was prepared with the ligation sequencing kit (SQK-LSK109, Oxford Nanopore Technologies) and was sequenced using an R9.4.1 flow cell on a GridION platform. The fast5 files were base called using Guppy, version 4.0.1, with the settings config dna_r9.4.1_450bps_hac and qscore_filtering; this generated 52,460 reads (883.7 Mb; *N*_50_, 44,301 bp). The Nanopore reads were *de novo* assembled using Flye, version 2.7 (6), with the following settings: nano-raw and genome-size 6M. This produced four contigs representing the two chromosomes and plasmids pVNTG1 and pVNTG2 (Table 1). Plasmids pVNTG3 and pVNTG4 were absent in the Flye assembly, but this was not surprising since the Oxford Nanopore Technologies ligation sequencing kit has been known to underrepresent small plasmids (7).

A total of six contigs, two from SPAdes assembly and four from Flye assembly, constituted the final assembly. The Illumina reads and Nanopore reads were aligned using minimap2 (8), version 2.17, with the settings ax sr for Illumina reads and ax map-ont for Nanopore reads. The resulting BAM files were used for polishing using HyPo (9), version 1.0.2, with the following settings: s 6m and c 50. We confirmed the circularity of the six contigs by visualizing the Nanopore read alignment using the Integrative Genomics Viewer (IGV), version 2.8.3 (10). The polished assembly was annotated on the DFAST server, version 1.2.4 (https://dfast.nig.ac.jp) (11).

The *V. nigripulchritudo* TUMSAT-TG-2018 genome consisted of two chromosomes and four plasmids, totaling 6,837,097 bp, with the overall GC content of 45.6% (Table 1). DFAST annotation predicted type IV secretion system components on plasmids pVNTG1 and pVNTG4, suggesting their role in virulence. A homology search using TBLASTN, version 2.11.0+, against the TUMSAT-TG-2018 genome found no match to nigritoxin (PDB ID 5M41), a bacterial toxin encoded by disease-associated *V. nigripulchritudo* strains from New Caledonia and Madagascar (2, 12).

### Data availability.

The TUMSAT-TG-2018 genome is available in DDBJ/EMBL/GenBank under the accession numbers AP024087 to AP024092. The raw read data are also available with the accession numbers DRR245513 and DRR245514.
